# Stickler syndrome – lessons from a national cohort

**DOI:** 10.1038/s41433-021-01776-8

**Published:** 2021-10-05

**Authors:** M. P. Snead, A. J. Richards, A. M. McNinch, P. Alexander, H. Martin, T. R. W. Nixon, P. Bale, N. Shenker, S. Brown, A. M. Blackwell, A. V. Poulson

**Affiliations:** 1 NHS England Highly Specialised Stickler Syndrome Diagnostic Service, Cambridge University NHS Foundation Trust, Addenbrooke’s Hospital, Hills Road, Cambridge, CB2 0QQ UK; 2 Vitreoretinal Research Group, John van Geest Centre for Brain Repair, University of Cambridge, Forvie Site, Robinson Way, Cambridge, CB2 0PY UK; 3 Department of Rheumatology Cambridge University NHS Foundation Trust, Addenbrooke’s Hospital, Hills Road, Cambridge, CB2 0QQ UK

**Keywords:** Risk factors, Mutation

## Abstract

In 2011 NHS England commissioned a new national specialist MDT service for patients and families affected by Stickler syndrome. The Stickler syndromes form part of the spectrum of inherited vitreoretinopathies and are the most common cause of retinal detachment in childhood and the most common cause of familial retinal detachment. Now in its 10th year, the Stickler Highly Specialised Service (HSS) has assessed 1673 patients from 785 families. Using a combination of accurate phenotyping and molecular genetic analysis it is possible to identify the underlying genetic mutation in over 95% of cases including those with deep intronic mutations likely to be missed by conventional exome panel analysis and which require whole gene sequencing and supplementary functional analysis to confirm pathogenicity. The vast majority that presents to ophthalmologists will be from one of three autosomal dominant sub-groups with a high associated risk of retinal detachment but the diagnosis is often overlooked, especially in adults. In contrast to many other blinding retinal conditions, blindness through giant retinal tear detachment particularly in children is largely preventable provided these high-risk groups are identified and appropriate evidence-based prophylaxis offered. This article summarises ten selected briefcase histories from the national dataset with key learning points from each.

## Introduction

In 2011 NHS England commissioned a new national specialist MDT service for patients and families affected by Stickler syndrome. The Stickler syndromes form part of the spectrum of inherited vitreoretinopathies and are the most common cause of retinal detachment in childhood and the most common cause of familial retinal detachment. Initially considered a mono-genic disorder, at least 10 clinically and genetically distinct sub-groups of Stickler syndrome are now recognised (Table 1) [1]. The principal clinical features are congenital myopia, retinal detachment, deafness, cleft palate and premature arthropathy.

**Table 1:** The Stickler syndromes and allied collagenopathies.

Syndrome		Gene	Cytogenetic location	Distinguishing features	Phenotype MIM No.
Stickler syndrome					
	Type 1	COL2A1	12q13.11	Type 1 Membranous congenital vitreous anomaly, retinal detachment, congenital megalophthalmos, deafness, arthropathy, cleft palate High risk of blindness	108300
	Ocular only	COL2A1	12q13.11	Type 1 Membranous congenital vitreous anomaly, retinal detachment congenital megalophthalmos. No systemic features. High risk of blindness	609508
	Type 2	COL11A1	1p21.1	Beaded type 2 congenital vitreous anomaly, retinal detachment, congenital megalophthalmos, deafness, arthropathy, cleft palate	604841
	Type 2 Recessive	COL11A1	1p21.1	Autosomal recessive, Beaded congenital vitreous anomaly, retinal detachment, congenital megalophthalmos, cleft palate, profound severe congenital deafness	*TBC*
	Type 3	COL11A2	6p21.32	Non-Ocular Stickler Normal vitreous and ocular phenotype, deafness, arthropathy, cleft palate	184840
	Type 4	COL9A1	6q13	Recessive inheritance, sensorineural deafness, myopia, vitreoretinopathy, retinal detachment, epiphyseal dysplasia	614134
	Type 5	COL9A2	1p34.2	Recessive inheritance, sensorineural deafness, myopia, vitreoretinopathy, retinal detachment, epiphyseal dysplasia	614284
	Type 6	COL9A3	20q13.33	Recessive inheritance, sensorineural deafness, myopia, vitreoretinopathy, retinal detachment, epiphyseal dysplasia	TBC
	Type 7	BMP4		Hypoplastic vitreous, retinal detachment deafness, arthropathy, palate abnormality, renal dysplasia	TBC
	Type 8	LOXL3	2p13.1	Recessive inheritance Congenital myopia, hypoplastic vitreous, palate abnormality, Arthropathy Normal facies Normal hearing	TBC
Kniest Dysplasia		COL2A1	12q13.11	(usually) Type 1 Membranous congenital vitreous anomaly, retinal detachment, congenital megalophthalmos, severe arthropathy, short stature, phalangeal dysplasia	156550
Spondyloepiphyseal dysplasia congenita (SEDC)		COL2A1	12q13.11	(usually) Type 1 Membranous congenital vitreous anomaly, retinal detachment, congenital megalophthalmos, severe short stature, Rhizomelic limb shortening, barrel chest	183900
Czech dysplasia		COL2A1	12q13.11	Hypoplastic vitreous, retinal detachment, cleft palate, normal stature, spondyloarthropathy, short postaxial toes	609162

Funded centrally the service provides MDT expertise free at point of care to all patients in England. The objectives are:

1. To provide accurate clinical and molecular genetic diagnosis and sub-classification of Stickler Syndrome for patients and families.

2. To develop a central patient registry and repository of data for longitudinal outcomes of all patients with Stickler syndrome in England to facilitate advancements in risk assessment, prophylaxis and treatment of the long-term complications of this disorder.

Now in its 10th year, the Stickler Highly Specialised Service (HSS) has assessed 1673 patients from 785 families. Using a combination of accurate phenotyping and molecular genetic analysis it is possible to identify the underlying genetic mutation in over 95% of cases so that prophylactic retinopexy can be offered to the high-risk sub-groups and reduce the risk of blindness [Table 1]. The last 10 years have also seen the emergence of an increasing array of sub-groups including ocular-only and autosomal recessive variants through a variety of molecular mechanisms [1].

This article summarises ten selected briefcase histories from the national dataset with key learning points from each.

## Case study 1

**Fig. 1:**
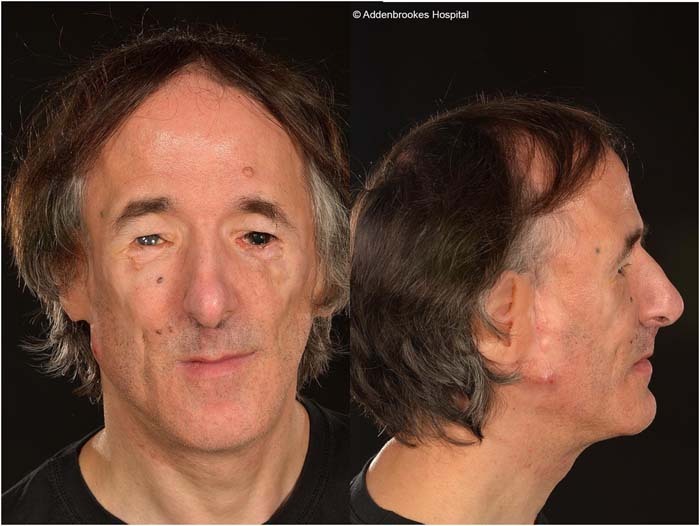
Case 1. Double heterozygosity – combined Treacher Collins syndrome inherited from father and type 2 Stickler syndrome inherited from mother. Type 2 Stickler syndrome vitreous phenotype (see Fig. 2).

The proband presented with Treacher Collins syndrome (TCS) at birth. Reconstructive operations were performed during childhood to correct facial anomalies which included cleft palate, micrognathia, microtia and maxillary hypoplasia (Fig. 1). Audiometry demonstrated mixed conductive and sensorineural hearing defects. The father, although not formally diagnosed with TCS, had malar flattening associated with bilateral lower lid malformations including bilateral agenesis of lower canaliculi and coloboma of the left lower lid.

**Fig. 2:**
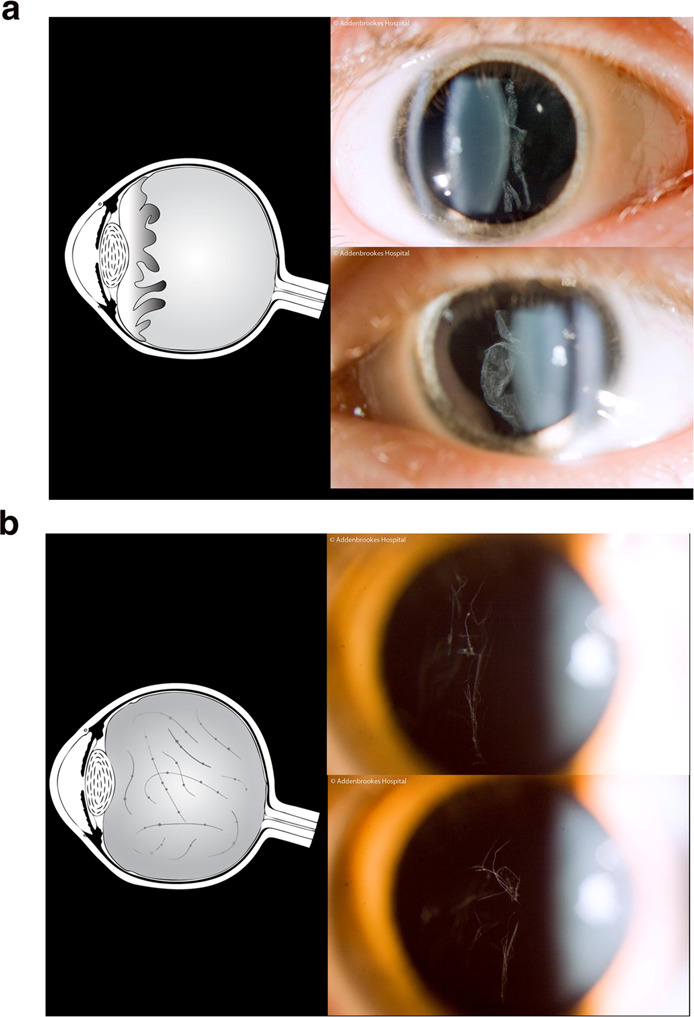
Vitreous phenotypes – pathognomonic of Stickler syndrome. Top 2a Schematic and slit-lamp illustration: Membranous congenital vitreous anomaly [Haploinsufficiency mutations COL2A1]. Bottom 2b. Schematic and slit-lamp illustration: Beaded congenital vitreous anomaly [COL11A1 dominant-negative mutations]. Reproduced with permission from [1].

The possibility of Stickler syndrome was not entertained until the age of 15 years when the proband attended the Stickler clinic as part of a screening process of maternal relatives for Stickler syndrome. His mother, uncle, maternal grandfather, and younger sister all exhibited the beaded vitreous phenotype characteristic of type 2 Stickler syndrome in association (Fig. 2b) with high myopia, midfacial hypoplasia, and mid-line clefting. The proband also exhibited the type 2 beaded vitreous phenotype (which is not a feature of TCS) and high myopia. Shortly after diagnosis, the patient developed left retinal detachment with multiple breaks which was successfully reattached. No prophylaxis was given to the fellow eye. Eight years later he developed a right retinal detachment with multiple breaks through 360 degrees which was successfully re-attached.

DNA analysis identified a single nucleotide substitution (c.1874G > T) in exon 19 of COL11A1, predicted to result in an amino acid substitution of a triple helix glycine (p.Gly625Val) in the α1 chain of type XI procollagen (type 2 Stickler syndrome).

### Key learning points

1. Vitreous phenotyping was key to the clinical diagnosis in an unusual compound phenotype, which was atypical for either disorder.
2. Risk of bilateral retinal detachment in Type 2 Stickler syndrome.

## Case study 2

The 20-month-old proband was referred from paediatric ophthalmology services with what was thought to be congenital cataract in both eyes. Both parents and his older sibling had normal vitreous phenotypes and there was no family history of cataract, retinal detachment or eye disease. On examination both eyes were hypotonous and the child was found to have right total cataract secondary to longstanding fixed funnel retinal detachment and also a total retinal detachment in fellow (left) eye but in which it was possible to visualise the membranous type 1 vitreous anomaly (Fig. 2a) when examined under anaesthesia. On the basis of the vitreous phenotype, fluorescent sequencing of COL2A1 was initiated and confirmed a heterozygous base change in exon 42 resulting in premature termination codon and haploinsufficiency typical of type 1 Stickler syndrome. Both right and left retinal detachments were successfully repaired but there has been no significant functional recovery of vision in the seven years of subsequent follow-up.

### Key learning points

1. Type 1 Stickler syndrome high risk of childhood blindness––in this case, bilateral Giant Retinal Tear detachments occurring in an infant under one year of age.
2. In sporadic cases, vitreous phenotype can be key in making diagnosis and directing subsequent laboratory analysis (Fig. 2a,b)

## Case study 3

The proband was aged 8 years and referred from paediatric ophthalmology services for consideration of surgery for an incidental finding of poor vision and unilateral cataract in his left eye. The proband had not reported or noticed the unilateral visual loss but had been documented to have a clear lens one year previously.

**Fig. 3:**
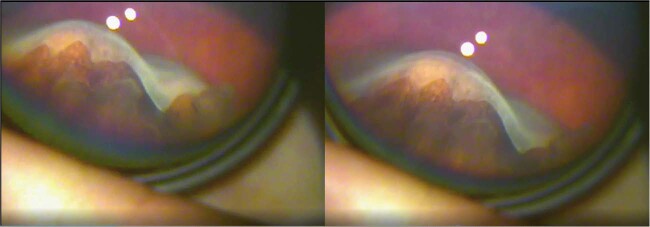
Case 3. 210 degrees Giant Retinal Tear identified at EUA in otherwise asymptomatic 8 yr old child.

Examination revealed, in addition to the membranous type 1 vitreous anomaly, secondary retinogenic lens epithelial metaplasia [2, 3] and heavy pigment dispersion in the vitreous cavity. During examination under anaesthesia he was found to have a peripheral retinal detachment with a 210 degrees Giant Retinal Tear in one eye and four peripheral tears in the fellow eye. (Fig. 3)

On the basis of the vitreous phenotype fluorescent sequencing of COL2A1 was initiated and confirmed a heterozygous deletion of two nucleotides in exon 52 of the COL2A1 gene resulting in a frameshift and haploinsufficiency, typical of type 1 Stickler syndrome.

### Key learning points

1. Type 1 Stickler syndrome carries a high risk of bilateral retinal detachment––incidental finding of a Giant Retinal tear in one eye and multiple retinal breaks in the fellow eye.
2. Examination under anaesthesia may be required to examine the peripheral retina to the ora serrata in a child.
3. Vitreous phenotype is key in making diagnosis and directing subsequent laboratory analysis.

## Case study 4

The proband was born with a cleft palate but no associated hearing loss and no family history of retinal detachment, cleft palate or deafness. At age three, he was found to be holding books very close and so-referred to ophthalmology services where cycloplegic refraction revealed bilateral highly myopic astigmatism (−11.00/−3.50 × 180) but was advised the risk of retinal detachment was “low”. Six months later he re-presented with an inoperable giant retinal tear detachment and despite surgery deteriorated to phthisis bulbi.

At age 6 he was referred by clinical genetics to the national Stickler Syndrome Diagnostic Service but because of severe persistent photophobia from his blind eye, slit-lamp examination was not possible. Targeted analysis of the known Stickler genes was carried out, failing to identify any pathogenic mutation in COL2A1, COL11A1, COL11A2, COL9A1, COL9A2, COL9A3 in the coding regions of these genes. An examination under anaesthesia was arranged which allowed visualisation of the type 1 membranous vitreous anomaly in the non-phthisical eye in addition to a 90-degree giant retinal tear which was successfully secured with 360 prophylactic cryoretinopexy. On the basis of the vitreous phenotype a diagnosis of Type 1 Stickler syndrome was made and a whole gene sequence analysis including all introns of COL2A1 was carried out which identified a deep intronic (c.2194-101 G > T in intron 33) sequence variant of unknown clinical significance. In silico analysis suggested it created a de novo donor splice site in the intron.

### Functional analysis

**Fig. 4:**
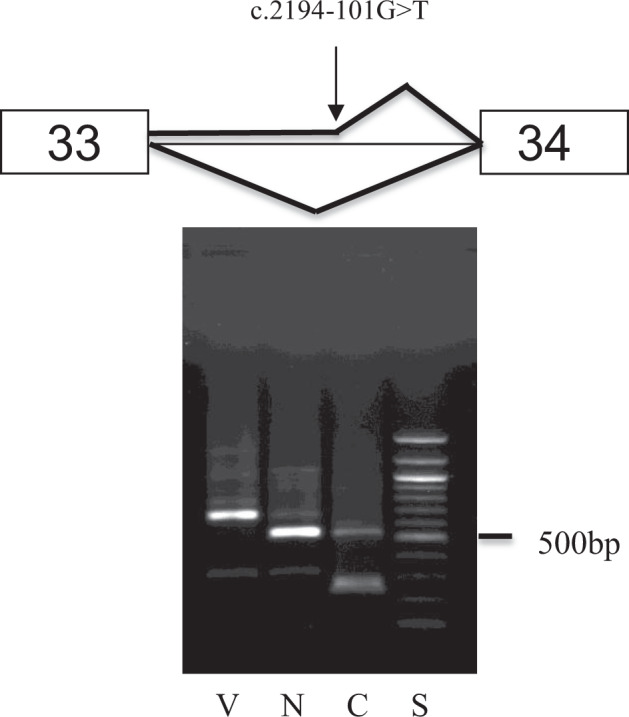
Case 4. Agarose gel electrophoresis showing that cDNA from the variant (V) minigene larger than that from the normal (N) minigene (left). cDNA sequencing showed that the variant contained extra sequence corresponding to intron 33 and use of a de novo donor splice site created by the variant, resulting in the addition of 136 nucleotides to the mRNA including a premature termination codon. Courtesy of Dr Allan Richards.

Both normal and variant DNA sequences were amplified and cloned to create minigenes, which were subsequently transfected and expressed in an ophthalmic cell line in vitro before harvesting and analysing RNA by RT-PCR. This showed that the variant cDNA contained extra sequence corresponding to intron 33 and use of a de novo donor splice site created by the c.2194-101 G > T variant which resulted in the addition of 136 nucleotides to the mRNA including a premature termination codon (Fig. 4).

### Key learning points

1. Deep intronic mutations may still be pathogenic, but require supplementary functional analysis to confirm pathogenicity.
2. Deep intronic mutations will be missed by conventional exome panel analysis–vitreous phenotyping was key to directing whole gene sequencing and supplementary mini-gene functional analysis
3. High risk of bilateral retinal detachment and blindness in Type 1 Stickler Syndrome. Prophylaxis is key to preserving vision in this sub-group.

## Case study 5

The patient was born with a cleft palate. She was moderately myopic and ocular examination demonstrated the membranous vitreous anomaly and a clinical diagnosis of type 1 Stickler syndrome were made. She did not demonstrate any signs of skeletal dysplasia. Neither parent demonstrated any clinical signs of Stickler syndrome. Genetic analysis confirmed a COL2A1 mutation, c.1597 C > T p.Arg533Ter in the child. Analysis of her clinically normal parents demonstrated a low level of the same mutation in the proband’s father. Quantitation by real-time PCR indicated that approximately 8% of the father COL2A1 alleles in lymphocyte DNA had the mutant sequence.

### Key learning points

1. New mutations are relatively common in Type 1 Stickler syndrome but mosaicism should be considered in apparently “de novo” cases [4].
2. Vitreous phenotype key to diagnosis in a case of cleft palate in the absence of both skeletal dysplasia and any family history of Stickler syndrome.

## Case study 6

**Fig. 5:**
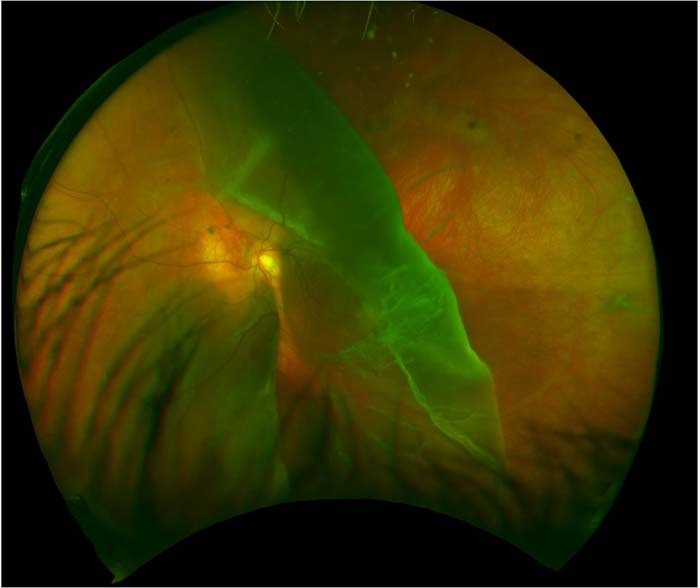
Case 6. GRT – child with undiagnosed type 1 Stickler syndrome, already blind in the fellow eye from retinal detachment. [GRT, giant retinal tear]. Reproduced with permission from [1].

The proband presented age 11, already blind in his right eye as a result of retinal detachment, erroneously ascribed to retinal dialysis. He was referred for advice about prophylaxis to his remaining eye. Examination of the fellow eye established the presence of the type 1 membranous vitreous anomaly consistent with type 1 Stickler syndrome and the diagnosis was confirmed on molecular genetic analysis. The rationale and objectives of prophylaxis to the fellow eye were discussed and the patient was listed for 360-degree prophylactic retinopexy according to standard published protocols [5, 6]. Unfortunately, surgery and theatre access were delayed by the COVID-19 pandemic and the child re-attended 5 months later with total loss of vision and Giant Retinal Tear detachment in his remaining eye (Fig. 5). His retinal detachment was successfully repaired but he has only regained 6/12- corrected vision to date.

### Key learning points

1. Retinal dialysis should not be confused with a Giant Retinal Tear (GRT) - the pathogenesis and surgical management are entirely different [7]. Although the oral disinsertion of a retinal dialysis may exceed 90° the vitreous is of normal architecture and characteristically remains attached to the posterior flap so that the independent mobility typical of a true giant retinal tear (GRT) is not a feature. Dialyses respond very well to conventional scleral buckling techniques [8] whereas a GRT will usually require an internal approach [9].
2. High risk of visual loss in Type 1 Stickler Syndrome. Although GRTs can be successfully repaired, prophylaxis rather than repair is key to preserving best vision in the high risk sub-groups (Table 1), [5, 6, 10, 11].
3. Half of all patients with genetically confirmed type 1 Stickler syndrome who experience retinal detachment suffer retinal detachment in their second eye within four years of the first eye [6].

## Case study 7

The proband was delivered by Caesarean section at 35 weeks and noted to have a cleft palate. There was a history of “blindness” in the paternal grandmother and a midwife mentioned that cleft palate and blindness “can sometime be associated”.

Two years later a full-term sibling was born by normal delivery and without palate abnormality.

At age four, the proband was noted by nursery staff to be holding books close and was found to be myopic at subsequent refraction. After searching the internet, the mother was concerned about possible Stickler syndrome in her eldest child and requested a referral to the national service.

Concurrently, the father developed floaters and sought advice at the local HES. No abnormality was detected on dilated fundal examination and the patient reassured and discharged. The possibility of Stickler syndrome volunteered by the patient and his wife was dismissed on the basis of a normal facial phenotype.

On attendance and examination at the National Stickler Clinic, vitreous phenotyping for the infants proved impossible but examination of the father confirmed the type 1 vitreous anomaly in addition to asymptomatic bilateral retinal detachments–a giant retinal tear in his right eye and a localised horse-shoe tear detachment in his left. Both retinal detachments were repaired.

At examination under anaesthesia both infants also exhibited the type 1 vitreous anomaly and both were found to have retinal tears at EUA. Fluorescent sequencing analysis of blood taken at EUA detected a heterozygous change of a single nucleotide (c.2896-1 G > A) at the intron 42/exon 43 boundary of the COL2A1 gene resulting in abnormal splicing of the COL2A1 mRNA (data not shown)

### Key learning points

1. Consider Stickler syndrome in any infant with cleft palate in association with myopia.
2. Affected adults, as well as children, are susceptible to GRT and prophylaxis should be considered in confirmed high risk cases irrespective of age.
3. Facial phenotype is unreliable for diagnosis exclusion–especially in adults and the ocular-only variants [1, 12, 13].
4. Examination of both parents may be valuable for any infant in whom Stickler syndrome is suspected but in whom vitreous examination is not possible.

## Case study 8

Two young boys both with severe deafness were referred. Neither parent was considered to show any signs of Stickler syndrome - their father had a high arched palate and mild, asymptomatic high tone hearing loss; the mother exhibited mild, asymptomatic hearing loss and normal vitreous. The proband had been born with congenital myopia (-7DS right and left), Pierre Robin sequence and profound hearing loss (90 dB), for which he had been given bilateral cochlear implants. His new-born brother (18 days old) also had Pierre Robin sequence, and auditory brainstem assessment demonstrated no recordable responses at over 95 dB.

Molecular genetic analysis of the gene for type 2 Stickler syndrome (COL11A1) identified 2 variants. One was clearly pathogenic–a frameshift mutation in exon 13; the other was a sequence variant (c.991-24 A > G) in intron 8 of unknown significance (single allele recorded on gnomAD v2.1.1, from 248,080 alleles sequenced).

*In silico* analysis of the second variant predicted the creation of an alternative exon 9 acceptor splice site. Functional mini-gene analysis showed that both the normal and mutant sequence produced a small amount of exon skipping, which reflected that exon 9 is alternatively spliced (expressed in different versions) in different tissues. However, the full length mutant cDNA appeared larger than the normal cDNA and contained an insertion of an additional 23 bp at the start of exon 9 which corresponded to the creation of a novel acceptor splice site, causing a shift in the reading frame and a downstream premature termination codon. Despite the normal acceptor splice site being present, functional minigene analysis showed that this was not being utilised. Because exon 9 is not expressed in all tissues, this mutation would be naturally removed from those tissues and have no effect, only being pathogenic in tissues where exon 9 is expressed.

### Key learning points

1. Recessive mutations in the COL11A1 gene normally result in fibrochondrogenesis––a severe (or even lethal) skeletal dysplasia.
2. This case demonstrates that effects of some recessive mutations in COL11A1 can be modified by alternative splicing and result in type 2 Stickler syndrome rather than fibrochondrogenesis. As with exon 2 in type 1 Stickler syndrome [12, 13] so the natural alternative splicing of COL11A1 exon 9 modifies the effect of such mutations reducing the severity of the associated skeletal dysplasia [14].
3. Exon 9 of COL11A1 is expressed in Meckel’s cartilage [15] which gives rise to the malleus and incus of the inner ear, in addition to the anterior ligament of the malleus tympanic plate [16] resulting in more severe hearing loss this sub-group of AR Stickler syndrome [14].
4. Disease phenotypes from *de novo* pathogenic variants can be modified by inherited recessive variants on the other allele [14, 17].

## Case study 9

The patient presented as the affected child of a mother already blind as a result of retinal detachment in both eyes. She underwent bilateral 360-degree prophylactic retinopexy at the age of 12 and has retained stable vision in both eyes over the subsequent 38 years.

She re-presented to the service aged 46 because of significant joint pain with reduced mobility and walking with a stick. She had had several steroid injections into her knee and hip joints with short lived effects. She had not found medications to be helpful. She had been working in a sedentary role but finding it difficult to attend work and was in danger of losing her job. Co-morbid depression was evident. X-rays demonstrated a skeletal dysplasia affecting her hips and knees with associated degenerative changes. She was recommended for joint replacement, starting with her hips.

A diagnosis of type 1 Stickler syndrome was made on the basis of vitreous phenotype and confirmed on molecular genetic analysis as a single base change at the exon 40/intron 40 boundary of the COL2A1 gene, c.2679 + 5 G > C, resulting in the abolition of the splice donor site leading to aberrant splicing.

### Key learning points

1. Patients with Stickler Syndrome can have skeletal abnormalities including a Marfanoid habitus, short stature and chest wall deformities.
2. The epiphyseal abnormalities most commonly cause femoral head changes in the hip and femoral condyle changes in the knee leading to early (4th-5th decade) joint arthroplasty. Other joints and the spine can also be affected. X-rays are indicated if there is joint pain.
3. Occasionally, platyspondyly (flattened vertebra) is misdiagnosed as vertebral fracture (osteoporosis).
4. No known disease modification is available but quality of life can be improved with pain management, including physiotherapy, and treatment of co-morbid sleep and mood disturbances
5. Early education and support are essential for optimal quality of life with appropriate education of employers and support services with regards to diagnosis and prognosis [18].

## Case study 10

The proband was referred aged three years with congenital high myopia, having undergone cleft palate repair at age one. A diagnosis of type 1 Stickler syndrome was made on the basis of vitreous phenotype and confirmed on molecular genetic analysis. Three-hundred-sixty degree prophylactic retinopexy was applied to both eyes and she progressed well in normal schooling and partook in various sporting activities including dancing and swimming. She developed right hip pain whilst undergoing Duke of Edinburgh Award training. Her examination demonstrated pes planus bilaterally with pain around her hip consistent with a soft tissue Greater Trochanteric Pain Syndrome. No X-ray was requested, given her age and likely diagnosis. She was advised to take analgesia (paracetamol and topical ibuprofen gel as necessary), supplied with tailored exercises, use over-the-counter medial arch insoles and continue with exercise. She completed her Award, cut back on weight-bearing activities and her pain settled.

### Key learning points

1. Joint hypermobility is more frequent in the Stickler Syndrome and is associated with pes planus and soft tissue pain, particularly around the ankle and knee. The wrist, base of thumb, spine and hip can also be affected. Dislocations can be associated with chronic pain.
2. The growing skeleton of the child affords an opportunity to correct disadvantageous biomechanics such as pes planus with education, orthotics and exercises.
3. Advice regarding sports should weigh up the social and health advantages with the risk of joint and ocular injury. Each case should be individually assessed. Usually, the advantages of any activity outweigh the risks.

## Summary

Although genetic conditions may be thought of as rare, Stickler syndrome is relatively common–the national service is seeing approximately 70 new index referrals per year. At least 10 clinically and genetically distinct sub-groups of Stickler syndrome are now recognised and with accurate phenotyping and molecular genetic analysis it is possible to identify the underlying genetic mutation in over 95% of cases. Deep intronic mutations may still be pathogenic, but will be missed by conventional exome panel analysis and will require whole gene sequencing and supplementary functional analysis to confirm pathogenicity. The vast majority that presents to ophthalmologists will be from one of three autosomal dominant sub-groups with a high associated risk of retinal detachment but the diagnosis is often overlooked, especially in adults. In contrast to many other blinding retinal conditions, blindness through giant retinal tear detachment particularly in children is largely preventable provided these high-risk groups are identified and appropriate evidence-based prophylaxis offered.

## Summary learning points

1. The NHSE national specialist MDT service is commissioned centrally and therefore “free” at point of care for patients and families affected by Stickler syndrome.
2. In addition to diagnosis and stratification of their risk of blindness it provides counselling and specialist adult and paediatric, rheumatology and audiology assessment for all patients as part of a one-stop MDT service.
3. Patients should be considered for referral to the service from the following categories:

(i) Infants with a history of congenital myopia in association with deafness
(ii) Infants born with cleft palate or Pierre Robin Sequence in association with myopia
(iii) Infants with joint hypermobility and/or epiphyseal dysplasia in association with myopia
(iv) Individuals suffering rhegmatogenous retinal detachment with a family history of rhegmatogenous retinal detachment.

4. Isolated cleft palate is not it itself an indication for referral unless associated with categories 3(i)-(iv) above.

## Future developments

NHSE central commissioning is just one example of the many ways in which the NHS leads the rest of the world in healthcare. To date, 1673 patients from 785 families with Stickler syndrome have been assessed as part of the NHSE Highly Specialised Service and well over 90% continue under annual review to help build an increasingly powerful database from which to evaluate treatments and outcomes of the long-term complications of this disorder.

For the laboratory molecular genetic analysis, deep intronic variants missed by conventional exome panel analysis may still be pathogenic. Next Generation Sequencing (NGS) can deliver rapid whole-genome sequencing, but does not solve the problem of determining the pathogenicity of such deep intronic variants. The NHSE HSS provides supplementary functional analysis to substantiate pathogenicity in such instances and is currently developing techniques by which this functional analysis can be enhanced.

### Summary

#### What is known about this topic

The Stickler syndromes form part of the spectrum of inherited vitreoretinopathies and are the most common cause of retinal detachment in childhood and the most common cause of familial retinal detachment.

#### What this study adds

The NHSE national specialist MDT service is commissioned centrally and therefore “free” at point of care for patients and families affected by Stickler syndrome. In addition to diagnosis and stratification of their risk of blindness it provides counselling and specialist adult and paediatric, rheumatology and audiology assessment for all patients as part of a one-stop MDT service. Patients should be considered for referral to the service from the following categories:

- Infants with a history of congenital myopia in association with deafness.
- Infants born with cleft palate or Pierre Robin Sequence in association with myopia.
- Infants with joint hypermobility and/or epiphyseal dysplasia in association with myopia.
- Individuals suffering rhegmatogenous retinal detachment with a family history of rhegmatogenous retinal detachment.
